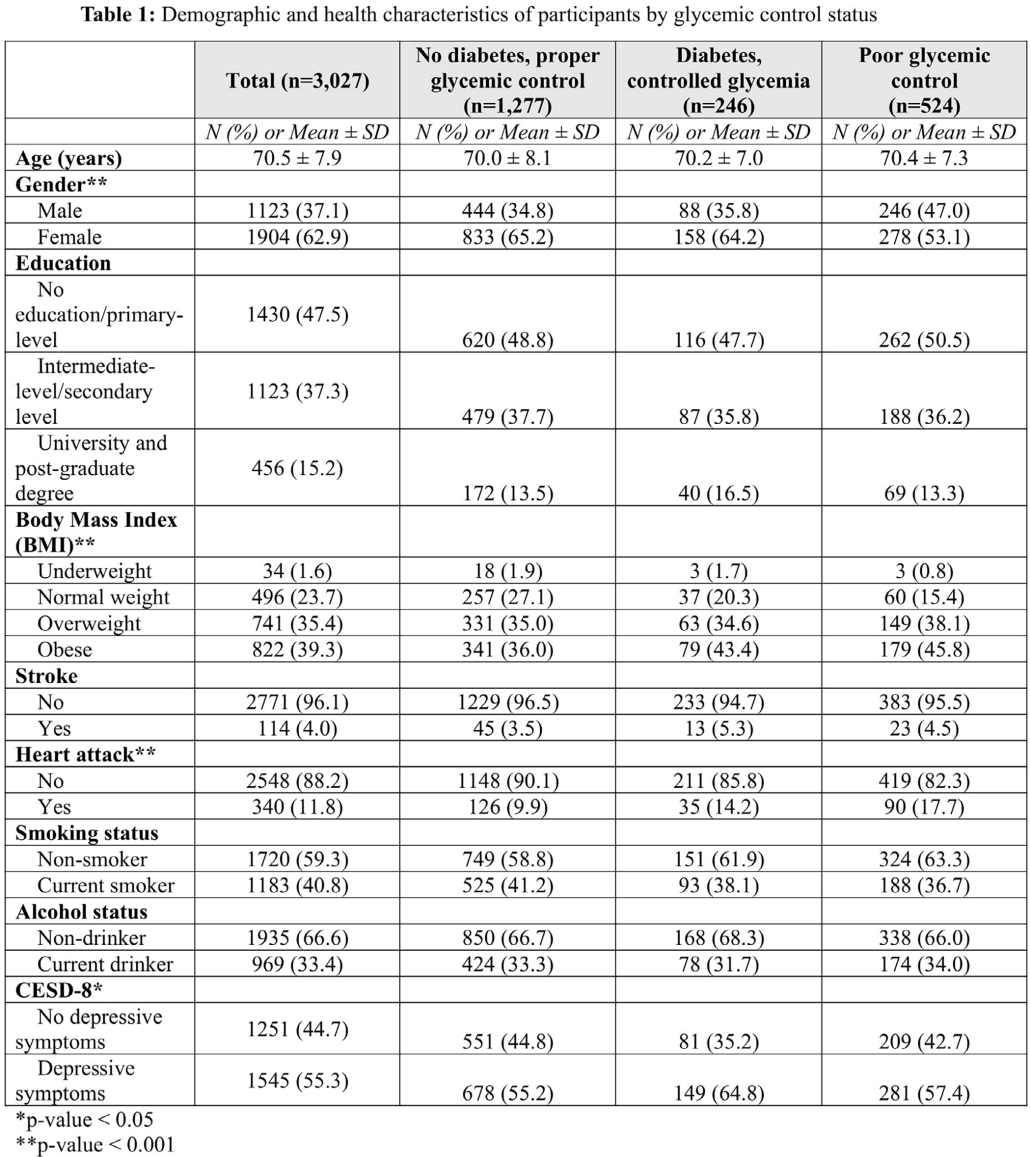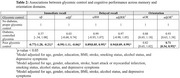# Association Between Glycemic Control and Affected Cognitive Domains Among Older Adults in Lebanon: A Population‐Based Cross‐Sectional Study

**DOI:** 10.1002/alz70860_106373

**Published:** 2025-12-23

**Authors:** Monique Chaaya, Aya El Sammak, Tanya El Khoury, Stephen McCall, Martine Elbejjani, Che Henry Ngwa, Carlos Mendes de Leon

**Affiliations:** ^1^ Faculty of Health Sciences, American University of Beirut, Beirut, Beirut, Lebanon; ^2^ Center for Research on Population and Health (CRPH), Beirut, Beirut, Lebanon; ^3^ Faculty of Medicine, American University of Beirut, Beirut, Lebanon; ^4^ Georgetown University, Washington DC, DC, USA

## Abstract

**Background:**

Type 2 diabetes mellitus is rising globally, with most of the burden found in low‐to‐middle income countries (LMIC). Diabetes is linked to cognitive decline, but the biological mechanisms and the role of glycemic control remains unclear. Conflicting results have been reported regarding the most affected cognitive domains and evidence from LMIC remains limited. This study aims to investigate the association between glycemic control and domain‐specific cognitive function among older adults in Lebanon.

**Method:**

Data were obtained from the Lebanon Study on Aging and HeAlth (LSAHA) (*n* = 3,027, mean age=70.5±7.9; 37% males), a population‐based, cohort study in urban and rural Lebanon. Older adults (60+) were selected using a multi‐stage, gridded sampling design. Glycemic control was categorized as follows: “No reported diabetes and proper glycemic control” (HbA1c < 6.5%); “reported diabetes and controlled glycemia” (HbA1c < 6.5%) and “poor glycemic control” (HbA1c > 6.5%, regardless of diabetes status). The association between glycemic control and cognitive performance was assessed across the following domains: memory (immediate and delayed recall) with linear and negative binomial regression, respectively; language (animal fluency) with linear regression; executive function (abstraction), orientation (time), and visuospatial (figure drawing) with logistic regression. Models were adjusted for age, gender, education, body mass index, stroke, heart attack, smoking, alcohol use, and depression.

**Result:**

Around 62% of participants had no diabetes and proper glycemic control, 12% had diabetes with controlled glycemia, and 26% had poor glycemic control. Only poor glycemic control was significantly associated with a ‐0.59 decrease (95%CI:[‐1.11,‐0.06]) in the immediate recall and an IRR of 0.94 (95%CI:[0.85,0.95]) in the delayed recall. It was also associated with lower odds of performing well in the orientation domain after adjustment (OR=0.71, 95%CI:[0.54,0.95]). No significant associations were found for language, visuospatial, or executive function.

**Conclusion:**

Results underscore the importance of glycemic control in cognitive performance. Participants with poor glycemic control exhibited marked lower scores in both immediate and delayed memory recall, and were less likely to perform well in orientation tasks. Effective diabetes management, particularly glycemic control, is essential to mitigate cognitive decline in aging populations.